# Impact of preoperative body mass index and weight loss on morbidity and mortality following colorectal cancer—a retrospective cohort study

**DOI:** 10.1007/s00384-022-04228-1

**Published:** 2022-08-11

**Authors:** Steffen Axt, Peter Wilhelm, Ricarda Spahlinger, Jens Rolinger, Jonas Johannink, Lena Axt, Andreas Kirschniak, Claudius Falch

**Affiliations:** 1 Department of General, Visceral and Transplant Surgery, Tübingen University Hospital, Hoppe-Seyler-Straße 3, 72076 Tübingen, Germany; 2 General and Visceral Surgery, Maria Hilf Hospital, Mönchengladbach, Germany; 3 Department of Internal Medicine I, Hospital Reutlingen, Steinenbergstr. 31, 72764 Reutlingen, Germany; 4 General and Visceral Surgery, Vorarlberg State Hospitals, Bregenz, Austria

**Keywords:** Preoperative body mass index, Preoperative weight loss, Colorectal cancer surgery, Risk factors

## Abstract

**Purpose:**

Body weight and preoperative weight loss (WL) are controversially discussed as risk factors for postoperative morbidity and mortality in colorectal cancer surgery. The objective of this study is to determine whether body mass index (BMI) or WL is associated with a higher postoperative complication rate.

**Methods:**

In this retrospective cohort study, data analysis of 1241 consecutive patients undergoing colorectal cancer surgery in an 11-year period was performed. The main outcome measures were wound infections (WI), anastomotic leakages (AL), and in-house mortality.

**Results:**

A total of 697 (56%) patients with colon and 544 (44%) with rectum carcinoma underwent surgery. The rate of WI for each location increased with rising BMI. The threshold value was 28.8 kg/m^2^. Obese patients developed significantly more WI than normal-weight patients did following rectal resection (18.0% vs. 8.2%, *p* = 0.018). Patients with preoperative WL developed significantly more AL following colon resections than did patients without preoperative WL (6.2% vs. 2.5%, *p* = 0.046). In-house mortality was significantly higher in obese patients following colon resections than in overweight patients (4.3% vs. 0.4%, *p* = 0.012). Regression analysis with reference to postoperative in-house mortality revealed neither increased BMI nor WL as an independent risk factor.

**Conclusions:**

Increased preoperative BMI is associated with a higher WI rate. AL rate after colon resection was significantly higher in patients showing preoperative WL. Preoperative BMI and WL are therefore risk factors for postoperative morbidity in this study. Nevertheless, this has to be further clarified by means of prospective studies.

**Trial registration** DRKS00025359, 21.05.2021, retrospectively registered.

## Background

Colorectal carcinomas are among the most prevalent carcinomas worldwide with an estimated number of new cases of about 1.1 million for colon and about 700,000 for rectal cancer, an estimated number of deaths of about 550,000 for colon, and about 310,000 for rectal cancer [1]. In the treatment of colorectal carcinoma, multimodal concepts depend on various influencing factors such as patient-specific factors (e.g., age, comorbidities), tumor-specific characteristics (e.g., stage, localization), treatment-associated factors (e.g., type of intervention, neoadjuvant therapy), and postoperative complications [2]. In colorectal surgery, risk factors of postoperative complications such as positive tobacco and alcohol anamnesis, age > 65 years, and existing comorbidities with an American Society of Anesthesiologists (ASA) score > III have already been determined [3, 4]. These are associated with higher treatment costs due to longer hospital stays, poorer functional and oncological outcomes, and increased mortality [3–8].

However, the influence of BMI is the subject of an ongoing controversial discussion [6, 9]. Also, the influence of unintentional WL in colorectal surgery on the postoperative complication rate is largely unexplored.

The objective of this retrospective cohort study is to determine the influence of preoperative BMI and WL on postoperative surgery-associated complications, specifically AL, WI, and in-hospital mortality (IHM).

## Methods

### Study design and setting

The study is a retrospective single-centered cohort study. All patients who underwent resection of colorectal carcinomas between the years 2004 and 2014 at the Department of General, Visceral and Transplant Surgery of Tübingen University Hospital were analyzed. Patients were identified by the hospital data system (I.S.H.*med; Siemens Medical Solutions GSD GmbH, Berlin; SAP SE; Walldorf, Germany) using DRG and OPS codes. A 1-year follow-up by means of standardized clinical data collection was added to the data analysis.

All oncological carcinoma resections including lymphadenectomy were performed in a standardized manner according to current evidence-based guidelines for colorectal cancer [10]. Carcinomas were evaluated as colon carcinomas up to the sigmoid colon. Carcinomas of the rectosigmoidal junction were classified as rectal carcinomas.

Multimodal therapies were administered in accordance with the recommendations of the interdisciplinary Center for Gastrointestinal Oncology of Tübingen University Hospital (Comprehensive Cancer Center).

BMI as measurement defines the quantitative variable “weight” statistically by relating body mass [kg] to height [m]. It is broken down by the World Health Organization into underweight (BMI < 18.5 kg/m^2^), normal weight (BMI 18.5–25 kg/m^2^), overweight (BMI 25–30 kg/m^2^), and obesity (BMI > 30 kg/m^2^) [11]. Unintentional WL as a dynamic variable can be seen as malnutrition. To rule out physiological weight fluctuations, in this study, preoperative WL was considered only if a documented, unintentional WL of at least 3 kg occurred in the last 6 months to achieve a weight loss of 5% according to the statistical average weight [12]. However, there is no uniform definition of preoperative WL in other studies [13–15].

Postoperative complications were classified in severity grades I–VI based on the Accordion Severity Classification [16], and comorbidities were graded with the Charlson Comorbidity Index [17]. Patients’ physical condition was classified according to the ASA score.

The main outcome measures were WI, AL, and IHM rates. WI were rated as a local inflammation with microbiological detection of pathogens [18] and AL with clinical and radiographic proof according to the literature [7, 9].

Categorization of quantitative variables into scores was performed for standardized data collection and better comparability. A confounding bias cannot be excluded from the study conducted. An adjustment of the effect was attempted to compensate by regression models.

### Statistical analysis

Statistical analysis was performed with SPSS® (IBM®, Armonk, NY, USA) using an Access database (Microsoft, Redmond, WA, USA).

The level of significance was determined with Fisher’s exact test (*n* > 60)/the Pearson chi^2^-test (*n* < 60) or the Mann–Whitney rank-sum test. To illustrate the relationship between metric and nominal values, a receiver operating characteristic (ROC) curve was compiled, and the Youden index was determined, if necessary. Logistic regression analysis was performed to determine the risk of occurrence of an event based on various factors. *p* values listed are two-sided, and *p* values < 0.05 are deemed statistically significant.

### Ethics

Ethics approval for the conduct of the study in compliance with the protection of the rights and welfare of human subjects participating in medical research (Ethics Review Board of the University of Tübingen, Germany; 273/2010BO2 and 379/2014BO2) was obtained and complies with the criteria of the STROBE checklist for cohort studies in surgery [19]. The study is registered in the German Clinical Trials Register (DRKS00025359). Informed consent has been obtained from the study participants.

### Cohort

A total of 1241 patients were recruited: 697 (56%) patients with colon and 544 (44%) with rectal carcinomas. Descriptive data analysis with patient population and differentiation of tumor entity distinguished by weight for colon and rectal carcinoma is presented in Tables 1, 2, 3, and 4.

**Table 1:** Demographics and postoperative complication rate for colon cancer referred to body mass index

	**Underweight**	**Normal weight**	**Overweight**	**Obesity**	**Total**
**Patient population (*n*/(%))**	20 (3)	296 (43)	255 (37)	115 (17)	686 (100)
**Age [year]**	67 (31–94)	66 (25–96)	68 (31–90)	68 (31–87)	67 (25–96)
**Gender (*n*/(%))**					
Male	4 (20)	134 (45)	159 (62)	70 (61)	367 (53)
Female	16 (80)	162 (55)	96 (38)	45 (39)	319 (47)
**CCI (points/(%))**					
0 points	7 (35)	111 (38)	74 (29)	22 (19)	214 (31)
1–2 points	5 (25)	61 (21)	77 (30)	37 (32)	180 (26)
> 2 points	8 (40)	124 (42)	104 (41)	56 (49)	292 (43)
**ASA score**^**x**^** (*n*/(%))**					
2	13 (65)	178 (63)	150 (62)	54 (49)	395 (59)
3	6 (30)	99 (35)	84 (35)	52 (47)	241 (36)
4	-	7 (2)	8 (3)	4 (4)	19 (3)
5	1 (5)	-	1 (< 1)	-	2 (< 1)
**Localization (*n*/(%))**					
Ascending colon	12 (60)	146 (49)	113 (44)	55 (48)	326 (48)
Transverse colon	2 (10)	24 (8)	25 (10)	9 (8)	60 (9)
Descending colon	3 (15)	39 (13)	33 (13)	11 (10)	86 (13)
Sigmoid colon	3 (15)	87 (29)	84 (33)	40 (35)	214 (31)
**UICC (*n*/(%))**					
Stage 0^y^	-	10 (3)	4 (2)	-	14 (2)
Stage I	2 (10)	45 (15)	52 (20)	23 (20)	122 (18)
Stage II	7 (35)	91 (31)	79 (31)	42 (37)	219 (32)
Stage III	4 (20)	64 (22)	60 (24)	21 (18)	149 (22)
Stage IV	7 (35)	85 (29)	60 (24)	29 (25)	181 (26)
**Resection (*n*/(%))**					
Right hemicolectomy	12 (60)	151 (51)	120 (47)	56 (49)	339 (49)
Transverse colon resection	2 (10)	11 (4)	11 (4)	2 (2)	26 (4)
Left hemicolectomy	4 (20)	45 (15)	34 (13)	14 (12)	97 (14)
Sigmoid colon resection	1 (5)	63 (21)	57 (22)	34 (30)	155 (23)
Multisegmental resection	1 (5)	26 (9)	33 (13)	9 (8)	69 (10)
**Approach (*n*/(%))**					
Open surgery	20 (100)	271 (92)	243 (95)	106 (92)	640 (93)
Laparoscopic surgery	-	25 (8)	12 (5)	9 (8)	46 (7)
Bridge-to-surgery	2 (10)	18 (6)	11 (4)	6 (5)	37 (5)
Emergency surgery	3 (15)	27 (9)	17 (7)	10 (9)	57 (8)
Simultaneous HIPEC	1 (5)	14 (5)	8 (3)	1 (< 1)	24 (3)
**Wound infection (*n*/(%))**					
Yes	-	20 (6.8)	23 (9.0)	11 (9.6)	54 (7.9)
No	20 (100)	276 (93.2)	232 (91.0)	104 (90.4)	632 (92.1)
**Level of wound infection (*n*/(%))**					
I°	-	14 (4.7)	13 (5.1)	9 (7.8)	36 (66.7)
II°	-	4 (1.4)	-	1 (0.9)	5 (9.3)
III°	-	1 (0.3)	4 (1.6)	-	5 (9.3)
IV°	-	1 (0.3)	5 (2.0)	1 (0.9)	7 (13.0)
V°	-	-	1 (0.4)	-	1 (1.9)
VI°	-	-	-	-	-
Total	-	20 (6.8)	23 (9.0)	11 (9.6)	54 (7.9)
**Anastomotic leakage (*n*/(%))**					
Yes	-	10 (3.4)	8 (3.1)	4 (3.5)	22 (3.2)
No	20 (100)	286 (96.6)	247 (96.9)	111 (96.5)	664 (96.8)
**Level of anastomotic leakage (*n*/(%))**					
I°	-	1 (0.3)	1 (0.4)	-	2 (9.1)
II°	-	-	-	-	-
III°	-	-	-	-	-
IV°	-	7 (2.4)	3 (1.2)	-	10 (45.5)
V°	-	2 (0.7)	3 (1.2)	2 (0.9)	7 (31.8)
VI°	-	-	1 (0.4)	2 (0.9)	3 (13.6)
Total	-	10 (3.4)	8 (3.1)	4 (3.5)	22 (100)
**In-hospital mortality (*n*/(%))**					
sMOF	1 (5.0)	4 (1.4)	1 (0.4)	5 (4.3)	11 (39.3)
CV/CP	1 (5.0)	8 (2.7)	4 (1.6)	4 (3.5)	17 (60.7)
Total	2 (10.0)	12 (4.1)	5 (2.0)	9 (7.8)	28 (4.1)

*CCI* Charlson Comorbidity Index, ASA score American Society of Anesthesiologists, *UICC* Union for International Cancer Control, *HIPEC* hyperthermic intraperitoneal chemotherapy, *sMOF* septic multi-organ failure, *CV/CP* cardiovascular/cardiopulmonary conditional organ failure

^x^percentages refer to 666 patients because it was undocumented in 31 patients

^y^UICC 0 was awarded for histopathological findings of a pTis

**Table 2:** Demographics and postoperative complication rate for colon cancer referred to preoperative weight loss

	**Preoperative weight loss**	**No preoperative weight loss**	**Total**
**Patient population (*n*/(%))**	130 (18.7)	567 (81.3)	697 (100)
**Age [year]**	67 (31–94)	67 (25–96)	67 (25–96)
**Gender (*n*/(%))**			
Male	69 (53)	305 (54)	374 (54)
Female	61 (47)	262 (46)	323 (46)
**CCI (points/(%))**			
0 points	35 (27)	183 (32)	218 (31)
1–2 points	35 (27)	148 (26)	183 (26)
> 2 points	60 (46)	236 (42)	296 (42)
**ASA score**^**x**^** (*n*/(%))**			
2	79 (66)	321 (59)	400 (60)
3	39 (33)	205 (36)	244 (37)
4	2 (2)	18 (3)	20 (3)
5	-	2 (< 1)	2 (< 1)
**Localization (*n*/(%))**			
Ascending colon	60 (46)	271 (48)	331 (48)
Transverse colon	12 (9)	49 (9)	61 (9)
Descending colon	17 (13)	70 (12)	87 (13)
Sigmoid colon	41 (32)	177 (31)	218 (31)
**UICC (*n*/(%))**			
Stage 0^y^	2 (2)	12 (2)	14 (2)
Stage I	10 (8)	113 (20)	123 (18)
Stage II	51 (40)	175 (31)	226 (33)
Stage III	24 (19)	126 (22)	150 (22)
Stage IV	43 (33)	140 (25)	183 (26)
**Resection (*n*/(%))**			
Right hemicolectomy	67 (52)	278 (49)	345 (49)
Transverse colon resection	3 (2)	23 (4)	26 (4)
Left hemicolectomy	24 (18)	75 (13)	99 (14)
Sigmoid colon resection	30 (23)	126 (22)	156 (22)
Multisegmental resection	6 (5)	65 (11)	71 (10)
**Approach (*n*/(%))**			
Open surgery	115 (88)	536 (95)	651 (93)
Laparoscopic surgery	15 (12)	31 (5)	46 (7)
Bridge-to-surgery	6 (5)	31 (5)	37 (5)
Emergency surgery	6 (5)	52 (9)	58 (8)
Simultaneous HIPEC	3 (2)	22 (4)	25 (4)
**Neoadjuvant therapy**			
Yes	11 (8.5)	15 (2.7)	26 (3.7)
No	119 (91.5)	552 (97.3)	671 (96.3)
**Wound infection (*n*/(%))**			
Yes	7 (5.4)	47 (8.3)	54 (7.7)
No	123 (94.6)	520 (91.7)	643 (92.3)
**Level of wound infection (*n*/(%))**			
I°	5 (3.4)	31 (5.5)	36 (66.7)
II°	-	5 (0.9)	5 (9.3)
III°	2 (1.5)	3 (0.5)	5 (9.3)
IV°	-	7 (1.2)	7 (13.0)
V°	-	1 (0.2)	1 (1.9)
VI°	-	-	-
Total	7 (5.4)	47 (8.3)	54 (100)
**Anastomotic leakage (*n*/(%))**			
Yes	8 (6.2)	14 (2.5)	697 (100)
No	122 (93.8)	553 (97.5)	22 (3.2)
**Level of anastomotic leakage (*n*/(%))**			
I°	1 (0.8)	1 (0.2)	2 (9.7)
II°	-	-	-
III°	-	-	-
IV°	4 (3.1)	6 (1.1)	10 (45.5)
V°	1 (0.8)	6 (1.1)	7 (31.8)
VI°	2 (1.5)	1 (0.2)	3 (13.6)
Total	8 (6.2)	14 (2.5)	22 (100)
**In-hospital mortality (*n*/(%))**			
sMOF	4 (3.1)	7 (1.2)	11 (37.9)
CV/CP	5 (3.8)	13 (2.3)	18 (62.1)
Total	9 (6.9)	20 (3.5)	29 (100)

*CC0I* Charlson Comorbidity Index, *ASA score* American Society of Anesthesiologists, *UICC* Union for International Cancer Control, *HIPEC* hyperthermic intraperitoneal chemotherapy, *sMOF* septic multi-organ failure, *CV/CP* cardiovascular/cardiopulmonary conditional organ failure

^x^Percentages refer to 666 patients because it was undocumented in 31 patients

^y^UICC 0 was awarded for histopathological findings of a pTis

**Table 3:** Demographics and postoperative complication rate for rectal cancer referred to body mass index

	**Underweight**	**Normal weight**	**Overweight**	**Obesity**	**Total**
**Patient population (*n*/(%))**	13 (2)	208 (38)	209 (39)	111 (21)	541 (100)
**Age [year]**	62 (42–77)	63 (19–91)	66 (38–86)	65 (34–88)	65 (19–91)
**Gender (*n*/(%))**					
Male	6 (46)	117 (56)	155 (74)	72 (65)	350 (65)
Female	7 (54)	91 (44)	54 (26)	39 (35)	191 (35)
**CCI (points/(%))**					
0 points	4 (31)	90 (43)	79 (38)	36 (32)	209 (39)
1–2 points	3 (23)	54 (26)	64 (31)	34 (31)	155 (29)
> 2 points	6 (46)	64 (31)	66 (32)	41 (37)	177 (33)
**ASA score**^**x**^					
2	6 (46)	164 (79)	154 (75)	67 (61)	391 (73)
3	7 (54)	43 (21)	48 (23)	40 (37)	138 (26)
4	-	-	4 (2)	2 (2)	6 (1)
5	-	-	-	-	-
**Localization (*n*/(%))**					
Rectosigmoid junction	3 (23)	26 (13)	33 (16)	20 (18)	82 (15)
Upper rectal third	4 (31)	42 (20)	54 (26)	20 (18)	120 (22)
Middel rectal third	3 (23)	89 (43)	86 (41)	39 (35)	217 (40)
Lower rectal third	3 (23)	51 (25)	36 (17)	32 (29)	122 (23)
**UICC (*n*/(%))**					
Stage 0^xx^	1 (8)	17 (8)	18 (9)	8 (7)	44 (8)
Stage I	-	56 (27)	54 (26)	33 (30)	143 (26)
Stage II	5 (39)	54 (26)	42 (20)	23 (21)	124 (23)
Stage III	2 (15)	41 (20)	53 (25)	28 (25)	124 (23)
Stage IV	5 (39)	39 (19)	42 (20)	19 (17)	105 (19)
**Resection**^**y**^** (*n*/(%))**					
Resection with primary anastomoses	11 (85)	160 (77)	172 (83)	85 (76)	427 (80)
Extirpation	2 (15)	47 (23)	34 (17)	26 (24)	109 (20)
**Approach**^**yy**^** (*n*/(%))**					
Open surgery	13 (100)	172 (83)	184 (88)	95 (86)	464 (87)
Laparoscopic surgery	-	34 (16)	22 (11)	15 (14)	71 (13)
Bridge-to-surgery	4 (31)	17 (8)	10 (5)	5 (4)	36 (7)
Emergency surgery	-	4 (2)	3 (1)	2 (2)	9 (2)
Simultaneous HIPEC	-	2 (1)	1 (< 1)	-	3 (< 1)
**Wound infection (*n*/(%))**					
Yes	2 (15.4)	17 (8.2)	25 (12.0)	20 (18.0)	64 (11.8)
No	11 (84.6)	191 (91.8)	184 (88.0)	91 (82.0)	477 (88.2)
Total	13 (2.4)	208 (38.4)	209 (38.6)	111 (20.5)	541 (100)
**Level of wound infection (*n*/(%))**					
I°	1 (7.7)	13 (6.3)	19 (9.1)	16 (14.4)	49 (76.6)
II°	1 (7.7)	1 (0.5)	3 (1.4)	1 (0.9)	6 (9.4)
III°	-	2 (1.0)	1 (0.5)	1 (0.9)	4 (6.3)
IV°	-	1 (0.5)	2 (1.0)	2 (1.8)	5 (7.8)
V°	-	-	-	-	-
VI°	-	-	-	-	-
Total	2 (15.4)	17 (8.2)	25 (12)	20 (18)	541 (100)
**Anastomotic leakage**^**z**^** (*n*/(%))**					
Yes	1 (9.1)	15 (9.4)	13 (7.6)	8 (9.5)	37 (8.7)
No	10 (90.9)	145 (90.6)	159 (92.4)	76 (90.5)	392 (91.8)
Total	11 (2.6)	160 (37.5)	172 (40.3)	84 (19.7)	427 (100)
**Level of anastomotic leakage (*n*/(%))**					
I°	-	2 (1.3)	2 (1.2)	1 (1.2)	5 (13.9)
II°	1 (9.1)	4 (2.5)	2 (1.2)	1 (1.2)	8 (22.2)
III°	-	2 (1.3)	2 (1.2)	-	4 (11.1)
IV°	-	7 (4.4)	7 84.1)	5 (6.0)	18 (50)
V°	-	-	-	-	-
VI°	-	-	-	1 (1.2)	1 (2.8)
Total	1 (9.1)	15 (9.4)	13 (7.6)	8 (9.5)	36 (100)
**In-hospital mortality (*n*/(%))**					
sMOF	-	1 (0.5)	-	1 (0.9)	2 (40)
CV/CP	-	2 (1.0)	-	1 (0.9)	3 (60)
Total	-	3 (1.5)	-	2 (1.8)	5 (0.9)

*CCI* Charlson Comorbidity Index, *ASA score* American Society of Anesthesiologists, *UICC* Union for International Cancer Control, *RWA* resection with primary anastomoses, *HIPEC* hyperthermic intraperitoneal chemotherapy, *sMOF* septic multi-organ failure, *CV/CP* cardiovascular/cardiopulmonary conditional organ failure

^x^Percentages refer to 535 patients because it was undocumented in 6 patients

^xx^UICC 0 was awarded for histopathological findings of a pTis and if histopathological examination after neoadjuvant therapy did not reveal any carcinoma

^y^Percentages refer to 536 patients because the type of resection was undocumented in 5 patients

^yy^Percentages in terms of surgical approach refer to 535 patients with 6 endoscopically resected carcinomas

^z^In 2 rectal carcinoma patients out of a total of 429 patients no classification of preoperative BMI groups could be made

**Table 4:** Demographics and postoperative complication rate for rectal cancer referred to preoperative weight loss

	**Preoperative weight loss**	**No preoperative weight loss**	**Total**
**Patient population (*n*/(%))**	79 (14)	465 (86)	544 (100)
**Age [year]**	66 (33–88)	64 (19–91)	65 (19–91)
**Gender (*n*/(%))**			
Male	48 (61)	305 (66)	353 (65)
Female	31 (39)	160 (34)	191 (35)
**CCI (*n*/(%))**			
0 points	28 (35)	181 (39)	209 (38)
1–2 points	21 (27)	136 (29)	157 (29)
> 2 points	30 (38)	148 (32)	178 (33)
**ASA score**^**x**^			
2	54 (69)	339 (74)	393 (73)
3	24 (31)	115 (25)	139 (26)
4	-	6 (1)	6 (1)
5	-	-	-
**Localization (*n*/(%))**			
Rectosigmoid junction	13 (17)	69 (15)	82 (15)
Upper rectal third	17 (25)	104 (22)	121 (22)
Middel rectal third	32 (41)	187 (40)	219 (40)
Lower rectal third	17 (22)	105 (23)	122 (22)
**UICC (*n*/(%))**			
Stage 0^y^	4 (5)	40 (9)	44 (8)
Stage I	14 (18)	130 (28)	144 (27)
Stage II	21 (27)	103 (22)	124 (23)
Stage III	16 (20)	110 (24)	126 (23)
Stage IV	24 (30)	81 (18)	105 (19)
**Resection**^**z**^** (*n*/(%))**			
Resection with primary anastomoses	63 (80)	366 (80)	429 (80)
Extirpation	16 (20)	93 (20)	109 (20)
**Approach**^**zz**^** (*n*/(%))**			
Open surgery	70 (90)	396 (86)	466 (87)
Laparoscopic surgery	8 (10)	63 (14)	71 (13)
Bridge-to-surgery	11 (14)	25 (5)	36 (7)
Emergency surgery	1 (1)	8 (2)	9 (2)
Simultaneous HIPEC	2 (3)	1 (< 1)	3 (1)
**Neoadjuvant therapy**			
Yes	42 (53.2)	219 (47.1)	261 (48)
No	37 (46.8)	246 (52.9)	283 (52)
**Wound infection (*n*/(%))**			
Yes	10 (12.7)	54 (11.6)	64 (11.8)
No	69 (87.3)	411 (88.4)	480 (88.2)
Total	79 (14.5)	465 (85.5)	544 (100)
**Level of wound infection (*n*/(%))**			
I°	8 (10.1)	41 (8.8)	49 (76.6)
II°	-	6 (1.3)	6 (9.4)
III°	1 (1.3)	3 (0.6)	4 (6.3)
IV°	1 (1.3)	4 (0.9)	5 (7.8)
V°	-	-	-
VI°	-	-	-
Total	10 (12.7)	54 (11.6)	64 (100)
**Anastomotic leakage (*n*/(%))**			
Yes	5 (7.9)	32 (8.7)	37 (8.7)
No	58 (92.1)	334 (91.3)	392 (91.4)
Total	63 (14.5)	366 (85.5)	429 (100)
**Level of anastomotic leakage (*n*/(%))**			
I°	2 (3.2)	3 (0.8)	5 (13.5)
II°	1 (1.6)	7 (1.9)	8 (21.6)
III°	-	4 (1.1)	4 (10.8)
IV°	2 (3.2)	17 (4.6)	19 (51.4)
V°	-	-	-
VI°	-	-	1 (2.7)
Total	5 (7.9)	32 (9.0)	37 (100)
**In-hospital mortality (*n*/(%))**			
sMOF	2 (0.4)	-	5 (100)
CV/CP	3 (0.6)	-	2 (60)
Total	5 (0.9)	-	3 (40)
**Diverting stomy (*n*/(%))**			
Yes	14 (6.5)	203 (93.5)	217 (50.6)
No	23 (10.8)	189 (89.2)	212 (49.4)
Total	37 (8.6)	392 (91.4)	429 (100)

*CC* Charlson Comorbidity Index, *ASA score* American Society of Anesthesiologists, *UICC* Union for International Cancer Control, *RWA* resection with primary anastomoses, *HIPEC* hyperthermic intraperitoneal chemotherapy, *sMOF* septic multi-organ failure, *CV/CP* cardiovascular/cardiopulmonary conditional organ failure

^x^Percentages refer to 535 patients because it was undocumented in 6 patients

^y^UICC 0 was awarded for histopathological findings of a pTis and if histopathological examination after neoadjuvant therapy did not reveal any carcinoma

^z^Percentages refer to 536 patients because the type of resection was undocumented in 6 patients and in 2 no classification of preoperative WL groups could be made

^zz^Percentages in terms of surgical approach refer to 535 patients with 7 endoscopically resected carcinomas

## Results

A total of 1241 patients underwent colorectal surgery in an 11-year period. Descriptive data analysis of the 697 (56.2%) treated colon carcinomas and the 544 (43.8%) rectal carcinomas referred to as preoperative BMI is described in Tables 1 and 3. Eleven patients with colon cancer and 3 patients with rectal cancer could not be categorized into a BMI group. However, weight loss was reported in these patients. In the case of colon carcinoma, preoperative WL was observed in 130 (19%) of the 697 patients and in the case of rectal carcinoma in 79 (15%) of the 544 patients (Tables 2 and 4).

### Rate of wound infection

As shown in Table 1, a total of 54 (7.9%) patients developed WI following colon resection. The rate of WI increased with higher BMI (normal weight: 6.8%; overweight: 9%; obesity: 9.6%). However, BMI was not seen to have a significant impact on the WI rate (*p* = 0.11) or WI severity.

Of the patients who underwent rectal resection, 64 (11.8%) developed WI (Table 3). A significant difference in WI rate was observed between normal weight and obese patients (*p* = 0.02). The ROC curve illustrates the influence of preoperative BMI on postoperative WI rate. A threshold level of 28.8 kg/m^2^ was detected. In abundance distribution, no significant difference was observed in the degree of severity relative to BMI.

Tables 2 and 4 show the influence of preoperative WL on the WI rate. After colon (*p* = 0.36) and rectal resection (*p* = 0.85), no significant difference in WI rate between patients with and without preoperative WL could be demonstrated as well as in the severity of WI.

### Rate of anastomotic leakage

As shown in Table 1, AL was diagnosed in 22 (3.2%) of the 686 patients following colon resection. The ROC curve showed no significant influence of preoperative BMI on the AL rate (*p* = 0.52), nor was a significant difference demonstrated with regard to the degree of severity.

In the 541 patients with rectal resection with primary anastomosis, AL was detected in 37 (8.7%) patients. A significant difference in the occurrence of AL among the different BMI groups could not be proven (*p* = 0.81). There was also no significant difference in the distribution of the severity of AL as shown in Table 3.

A significant difference in AL rate following colon resection between patients with and without preoperative WL was demonstrated (*p* = 0.046). There were no significant differences in the severity of AL (Table 2).

After rectal resection, an AL rate of 8.7% (37 out of 429) was observed with no significant difference between patients with and without preoperative WL (*p* = 0.81) nor was a significant difference in the severity of AL observed (Table 4). Moreover, no significant difference (*p* = 0.2) was seen between patients with an ileostomy (*n* = 14; 6.5%) and without an ileostomy (*n* = 23; 10.8%).

### Rate of in-hospital mortality

IHM following colon resection referred to preoperative BMI was 4.1% (*n* = 28), caused by multi-organ failure as a result of sepsis in 39.3% (*n* = 11) and cardiovascular or cardiopulmonary events in 60.7% (*n* = 17). There was a significant difference in IHM from multi-organ failure as a result of sepsis between overweight and obese patients (*p* = 0.01), as shown in Table 1. No further significant differences were detected between the other BMI groups.

IHM following rectal resection referred to preoperative BMI was 0.9% (*n* = 5), caused by multi-organ failure as a result of sepsis in 40% (*n* = 2) and cardiovascular or cardiopulmonary events in 60% (*n* = 3) (Table 3). Mortality affected only normal weight and obese patients.

IHM following colon and rectal resection referred to as preoperative weight loss showed no significant differences between the two groups with regard to multi-organ failure as a result of sepsis or cardiovascular or cardiopulmonary events.

### Regression analysis

Regression analysis as shown in Table 5 demonstrated that Charlson Comorbidity Index, ASA score, UICC stage, age, and emergency surgery are factors exerting a significant influence on IHM. With an increasing ASA score, the risk of IHM rises significantly (*p* = 0.049). An increase of one score point doubles the risk of IHM (OR 2.008; 95% CI: 1.003–4.021). With an increase in Charlson Comorbidity Index, the risk of IHM rises significantly, namely by 34% per score point (OR 1.340; 95% CI: 1.157–1.553; *p* = 0.001). Patients with UICC IV also had a significantly increased risk of IHM as compared to stages 0–I (OR 3.228; 95% CI: 1.014–10.283; *p* = 0.047). Age (OR 1.072; 95% CI: 1.020–1.126; *p* = 0.006) and emergency surgery (OR 0.126; 95% CI: 0.047–0.334; *p* = 0.001) were seen to have an increased risk for IHM. Regression analyses of the incidence of postoperative WI and AL were not reliable due to the small numbers of cases.

**Table 5:** Factors influencing in-hospital mortality in a multivariable regression analysis

Influencing factors	Odds ratio (OR) [95% CI]	*p*-value
Preoperative BMI	1.028 [0.946–1.116]	0.52
Preoperative weight loss	0.595 [0.219–1.617]	0.31
Postoperative appearance of WI	1.697 [0.406–7.093]	0.47
Postoperative appearance of AL	0.361 [0.092–1.425]	0.15
Localization		
Colon	1	
Rectum	1.742 [0.606–5.004]	0.30
ASA score	2.008 [1.003–4.021]	0.049
CCI	1.340 [1.157–1.553]	0.001
UICC		
0–I	1	
II–III	2.174 [0.450–10.506]	0.33
IV	3.228 [1.014–10.283]	0.047
Age	1.072 [1.020–1.126]	0.006
Emergency operation	0.126 [0.047–0.334]	0.001

*BMI* body mass index, *WI* wound infection, *AL* anastomotic leakage, *ASA score* American Society of Anesthesiologists, *CCI* Charlson Comorbidity Index, *UICC* Union for International Cancer Control

## Discussion

In our retrospective study of 1241 patients who underwent colorectal cancer surgery in an 11-year period median age [20], sex ratio [21], UICC stages [22], surgical techniques [23], rate of emergency surgery [24], and preoperative BMI [25] were similar compared to other studies. Normal weight patients constituted the largest group in this study population. Compared to the weight distribution of the average population underweight, overweight and obese patients were disproportionately represented [12]. Even though BMI is uncomplicated to determine clinically and well compared to other studies, it is critically discussed in the literature due to its nonuniform use. For example, BMI ≥ 25 kg/m^2^ was rated overweight or obese without considering other factors such as muscle mass [26]. Other parameters, such as waist-to-size ratio [15], waist circumference, waist-to-hip ratio, or visceral fat, are described as being more sensitive [27]. Nevertheless, BMI is the value most frequently used in publications so that it was also chosen as a reference in the present study.

Of the patients in our study, 17% (*n* = 209) presented with preoperative WL, which is comparable to other studies with 7–28% [28, 29]. Unintentional WL as a dynamic value is dealt with variously by different studies and can be seen as malnutrition. The pathophysiology of unintentional WL is poorly understood. Malignant diseases are cited as the most frequent cause of unintentional WL prior to nonmalignant gastrointestinal diseases, psychiatric conditions, and unknown causes [13, 30, 31].

Our study demonstrated a significant influence of neoadjuvant therapy for colon carcinoma on preoperative WL (*p* = 0.004) (Table 2). However, the small number of this subgroup should be taken into account (26/697; 3.7%). In rectal carcinoma, 48% of the patients received neoadjuvant therapy, of which 53.2% presented with preoperative WL. Here, neoadjuvant therapy had no significant influence on preoperative WL (*p* = 0.33) (Table 4).

The WI rate following colon and rectal resection increased with rising BMI. After colon resection, the WI rate increased significantly in patients with BMI ≥ 30 kg/m^2^ as compared to patients with BMI ≤ 30 kg/m^2^. Underweight patients did not develop WI following colon resection in either this study or other studies [18].

After rectal resection, obese patients developed significantly more WI than normal weight patients did. This is comparable to other study results [32, 33]. The threshold value for developing WI after rectal resection was seen to be 28.8 kg/m^2^. There are no comparable threshold values available.

Patients in our study with or without preoperative WL showed no significant difference in the WI rate following a colon or rectal resection. An increased WI rate caused by WL from malnutrition proved to be an independent risk factor [34]. Furthermore, the possibility of lowering the WI rate for WL by using immunonutrition has already been described [35]. However, another study performed with preoperative immunonutrition therapy showed no change in the postoperative complication rate when bloodwork improved [36]. In contrast to our study, Tang et al. showed an increased WI rate after preoperative WL [37]. Evidence on the influence of preoperative WL on the WI rate following colon and rectal resection is therefore inconsistent.

With regard to AL following a colon or rectal resection, no significant differences between the different BMI groups were found in our study. The highest rate of AL was seen in the group of obese patients but without significant difference. Nevertheless, the evidence is inconsistent. Gessler et al. did not report any significant difference in a retrospective evaluation of 600 patients [7]. In retrospective national analyses, Midura et al. demonstrated no significant difference in the AL rate between obese (BMI > 30 kg/m^2^) and nonobese patients in a collective of 13,684 patients [8] as well as Bakker et al. in a collective of 15,667 patients [38]. In contrast, Qiu et al. conducted a meta-analysis of ten studies with a total of 3660 obese and 10,829 nonobese patients and demonstrated a significantly increased risk of AL in patients who already had a BMI > 25 kg/m^2^ (8% versus 2.2%; *p* < 0.001) [39]. This was also evident in Amri et al. [18]. Geiger et al. pointed out a significant difference in the AL rate following colon resection between nonobese (BMI < 30 kg/m^2^) and obese patients (BMI > 30 kg/m^2^). That study did not discriminate between colon and rectal carcinoma [40]. The AL rate following rectal resection ranged from 7.6 to 9.5% for the various BMI groups. Benoist et al. as well as Qu et al. in a meta-analysis of 1619 prospectively and 2967 retrospectively evaluated patients identified obesity as a risk factor for AL following rectal resection [6, 41]. As possible reasons for difficult operative conditions, technical difficulties due to visceral obesity and unclear anatomical conditions were discussed [6, 33, 42]. Following colon resection, there was a significant increase in the AL rate in patients with preoperative WL. This was also demonstrated by Rencuzogullari et al. in patients > 65 years after elective resections [43]. After rectal resection, a more likely occurrence of AL was demonstrated in patients with preoperative WL than in those without, however with no significant difference. In contrast, Kang et al. showed a significantly increased AL rate following rectal resection in patients with preoperative WL [44]. Midura et al. also demonstrated a significantly higher rate of AL in patients compared to patients without WL [8]. Like the influence of BMI on the AL rate, the influence of preoperative WL also needs to be further discussed in different data situations.

Following colon resection, obese patients died significantly more frequently than did overweight patients due to septic multiorgan failure. This was also demonstrated by Amri et al., who, however, did not discriminate between colon and rectal carcinoma [18]. Hu et al. pointed out that 5.9% underweight patients had a significantly higher 30-day mortality rate than did non-underweight patients [45].

Following rectal resection, no significant difference in mortality rate was demonstrated between the different BMI groups. Here, Hu et al. demonstrated also a higher mortality rate after rectal resection in underweight patients [45]. In our study, however, no underweight patient died after rectal resection. Bakker et al. did not demonstrate a correlation between increased BMI and AL or associated higher mortality [38].

Following colon resection, significantly more patients with preoperative WL died than did patients without preoperative WL. After rectal resection, no patient with preoperative WL died. The mortality rate in patients without preoperative WL was also very low, namely 0.9%. Midura et al. were able to demonstrate a significant correlation between preoperative WL and AL. They also demonstrated a correlation between AL and the 30-day mortality rate, which was significantly increased. Thus, it can be concluded that preoperative WL influences the 30-day mortality rate [8]. To further determine the influence of BMI and preoperative WL on the 30-day mortality rate, further studies are needed to clarify the inconsistent data.

Significant differences in IHM following colon and rectal resection between the two groups were not demonstrated in our study. This was supported by regression analysis that revealed Charlson Comorbidity Index, ASA score, UICC stage, age, and emergency resection to be significant influencing factors for IHM. Neither preoperative BMI, WL, nor the occurrence of WI or AL showed a significant influence. In other studies, a higher mortality rate following colorectal cancer surgery was pointed out in patients with comorbidities, higher UICC stages, age > 65 years, and obesity [17, 24, 45]. Regression analysis with reference to postoperative IHM revealed neither increased BMI nor WL as an independent risk factor.

Limitations of the present study include the retrospective study design and the difficult comparability with the data of other studies as a consequence of different BMI and WL definitions. Also, selection and information bias have to be pointed out in the context of retrospective data evaluation. Postoperative complications after colorectal cancer surgery are multifactorial so that a confounding bias of the individual complications cannot be excluded.

Nevertheless, it must be pointed out that despite retrospective data collection, the complete data sets of an 11-year period could be evaluated. A strict distinction between entities of colon and rectum was performed. This resulted in high case numbers of the two collectives in our study. Both preoperative BMI and WL represent a risk factor of increased morbidity in colorectal cancer surgery in our study.

## Conclusions

Obese patients were seen to have a significantly higher WI rate than normal weight patients following rectal resection. There was also a significant increase in the AL rate following colon resection in patients with preoperative WL. IHM was significantly increased in obese patients as compared to overweight patients following colon resection. Nevertheless, in multivariable regression analysis, neither preoperative BMI nor WL was an independent risk factor for increased IHM.

While the reasons for WI, AL, and IHM are multifactorial, they are not independent risk factors for the occurrence of these surgery-associated complications. With a simple evaluation of these factors, they could possibly be components of preoperative risk stratification. This should be further investigated in prospective randomized controlled trials. The studies available to date are inhomogeneous and controversial.

## Data Availability

The datasets used and analyzed during the current study are available from the corresponding author on reasonable request. Further study data can also be accessed in the German Clinical Trials Register (DRKS00025359).
